# Fabrication of Yolk-Shell Cu@C Nanocomposites as High-Performance Catalysts in Oxidative Carbonylation of Methanol to Dimethyl Carbonate

**DOI:** 10.1186/s11671-017-2258-7

**Published:** 2017-08-08

**Authors:** Juan Wang, Panpan Hao, Ruina Shi, Leilei Yang, Shusen Liu, Jinxian Zhao, Jun Ren, Zhong Li

**Affiliations:** Key Laboratory of Coal Science and Technology (Taiyuan University of Technology), Ministry of Education and Shanxi Province, No. 79 Yingze West Street, Taiyuan, 030024 China

**Keywords:** Confinement effect, Cu@C nanocomposites, Yolk-shell structures, Oxidative carbonylation, Dimethyl carbonate

## Abstract

A facile way was developed to fabricate yolk-shell composites with tunable Cu cores encapsulated within hollow carbon spheres (Cu@C) with an average diameter about 210 nm and cavity size about 80 nm. During pyrolysis, the confined nanospace of hollow cavity ensures that the nucleation-and-growth process of Cu nanocrystals take place exclusively inside the cavities. The size of Cu cores can be easily tuned from 30 to 55 nm by varying the copper salt concentration. By deliberately creating shell porosity through KOH chemical activation, at an optimized KOH/HCS mass ratio of 1/4, the catalytic performance for the oxidative carbonylation of methanol to dimethyl carbonate (DMC) of the activated sample is enhanced remarkably with TOF up to 8.6 h^−1^ at methanol conversion of 17.1%. The activated yolk-shell catalyst shows promising catalytic properties involving the reusability with slight loss of catalytic activity and negligible leaching of activated components even after seven recycles, which is beneficial to the implementation of clean production for the eco-friendly chemical DMC thoroughly.

**Electronic supplementary material:**

The online version of this article (doi:10.1186/s11671-017-2258-7) contains supplementary material, which is available to authorized users.

## Background

Dimethyl carbonate (DMC) has attracted much attention as a widely used building block due to its excellent biodegradability (e.g., low bioaccumulation and persistence) and low toxicity [1]. The potential industrial applications of DMC cover many fields, such as nonpoisonous solvent, alternative substitute for phosgene, fuel additive and intermediate for the synthesis of polycarbonates and isocyanates [2–5]. In view of various synthetic method of DMC, the oxidative carbonylation of methanol (MeOH) using CO, O_2_, and MeOH as raw materials has been representing one of the proposed favorable process owing to the high utilization rate of carbon source and environmental benefits. The catalysts used in this reaction can mainly be classified into two types: chlorine-containing catalysts and chlorine-free ones. Since there are some problems, such as severe corrosive problems, deteriorate product quality, and catalyst deactivation, that stem from the loss of chlorine from the chlorine-containing catalysts, chlorine-free catalysts have been extensively studied [6, 7]. Activated carbon (AC) supported copper or copper oxide have been shown promising catalytic activity for DMC synthesis [8–10], and researchers have suggested that Cu is the active center for this reaction [10–13]. However, the deactivation of supported copper catalysts are generally attributed to the agglomeration of copper particles, loss of active species, and change of copper’s chemical state, among which, the former is more serious. In order to overcome such drawbacks, the design and fabrication of nanoparticle encapsulated into a protective shell is beneficial for reinforcing the catalytic activity and stability of reactive centers in the oxidative carbonylation of methanol to DMC from the technological point of view.

Along this line, yolk-shell nanostructures (YSNs) or rattle-type nanocomposites, in which core nanoparticles (NPs) are encapsulated by an outer layer with an interstitial free space between them, have been particularly popular due to their unique hierarchical/multilevel nanostructures, and accompanying optical and electrical properties and great potential in catalytic application [14]. The protective shell in YSNs can effectively keep the core element stable even under harsh conditions and sufficiently expose its active surface [15]. The enclosed void space is expected to be useful for chemical storage, compartmentation, and confinement of host-guest interactions, and more importantly, providing a unique environment for creating concerted actions between the core and a permeable shell [16]. These remarkable textural characteristics enable YSNs to function as promising candidate to satisfy the demands like sinter-stable and reusability for applications in catalysis. Among them, yolk-carbon shell nanostructures have immediately attracted considerable interest owing to the inherent conductivity as well as excellent chemical and thermal stability of the carbon coating [17–21].

Recently, Lu and co-works have reported the preparation of hollow spheres through weak acid–base interaction-induced assembly with the use of oleic acid a soft template and functional dihydroxybenzoic acid (DA) as precursor [22]. Herein, we extend their work to develop a facile towards the YSNs with tunable Cu core size encapsulated inside hollow carbon spheres (HCSs) (Cu@C) by employing a ship-in-bottle strategy. The shell porosity of Cu@C heterogeneous catalysts can be tuned by KOH activation, and its effects on the catalytic performances and stability in DMC synthesis are also investigated.

## Methods

### Chemicals

2,4-Dihydroxybenzoic acid (DA) was obtained from J&K Scientific Ltd. Oleic acid, ammonia solution (25%), formaldehyde, copper nitrate (Cu(NO_3_)_2_·3H_2_O), potassium hydroxide (KOH), and methanol (MeOH) were obtained from the Sinopharm Chemical Reagent Co. Ltd. All chemicals were of analytical grade and used without any further purification. Deionized water obtained from Milli-Q system (Millipore, Bedford, MA) was used in all experiments. O_2_ (>99.99%) and CO (>99.99%) were supplied by the Beijing ZG Special Gases Science & Technology Co. Ltd.

### Synthesis of Hollow Carbon Spheres (HCS)

The hollow polymer spheres (HPSs) with a hollow core and a polymer shell were first prepared using oleic acid as soft template and phenolic resin as carbon precursor following the procedure reported by Lu et al. [22]. In a typical procedure, 2.5 mmol of 2,4-dihydroxybenzoic acid and 7.5 mmol of formaldehyde were dissolved in 95 mL of deionized water. A 5-mL volume of an aqueous solution containing 56 μL of oleic acid and 180 μL of ammonia solution (25%) was added to the above-prepared solution at 30 °C under slow stirring for 30 min. Next, the mixture was transferred into an autoclave hydrothermally aged for 4 h at 140 °C. After centrifugation, washed with deionized water and ethanol, dried at 50 °C overnight, and then pyrolyzed at 700 °C for 2 h under a nitrogen flow, the HCS was obtained.

### Synthesis of Cu@C Nanocomposite Materials

Typically, 0.3 g of the as-prepared HCSs was first dispersed in 30 mL of copper nitrate solution with different concentration range from 0.03 to 0.24 M. Then, the mixture was transferred into an autoclave to undergo a hydrothermal impregnation at 100 °C for 10 h. The resulting impregnated sample, denoted as HCS-Cu^2+^, was retrieved by the same method as HPS. After calcined at 400 °C for 2 h under H_2_/N_2_ (10%/90%), finally, the yolk-shell Cu@C-X (X = 0.03, 0.06, 0.12, 0.24) nanocomposites were obtained.

### Synthesis of Cu@A-HCS Catalyst with KOH-Activated Carbon Sphere as Support

The treatment of HCS with KOH is attempted, with the intent of modifying the characters of carbon support and further affecting the performance of Cu catalyst. Typically, 0.3 g HCSs were mixed with 0.15 g KOH physically in the absence of water. After the pre-treatment, the sample was heated in 80 mL/min nitrogen stream with a ramp rate of 10 °C/min up to 700 °C for 2 h and then cooled to room temperature. The KOH post-treated carbons were washed repeatedly with diluted HCl and subsequently with distilled water until no chlorine ion was detected (AgNO_3_ test). After dried at 60 °C overnight, 0.12 M copper nitrate solution was used during the hydrothermal impregnation and other procedures were identical to that of Cu@C-0.12, finally yielding the modified samples denoted as Cu@A-HCS.

### The Catalytic Performance of Cu@C-X (X = 0.03, 0.06, 0.12, 0.24) and Cu@A-HCS

Oxidative carbonylation of methanol was carried out in a 25-mL stainless steel autoclave lined with Teflon and equipped with a magnetic stirrer. In a typical experiment, 0.2 g catalyst and 10 mL methanol were loaded into the autoclave, which was then sealed tightly, purged three times with CO and next pressurized to 3.0 MPa with CO and O_2_ (P_CO_:P_O2_ = 2:1) at room temperature. The reaction proceeded at 120 °C with continuously stirring at 750 rpm for 1.5 h. After the reaction, the reactor was cooled down to the room temperature and depressurized. The catalysts were separated by filtration. The concentrations of products in filtrate were determined by gas chromatography (GC) using an FID detector. The recyclability of the used catalyst was studied by performing a series of consecutive runs.

The main reaction of the oxidative carbonylation of methanol to dimethyl carbonate was shown as below:

2CH_3_OH + 1/2 CO + O_2_ = (CH_3_O)_2_CO + H_2_O.

The concentration of copper, MeOH conversion (C_MeOH_), DMC selectivity (S_DMC_), and Turnover frequency (TOF) were calculated by the following equations:

The concentration of copper (C_Cu_, mmol/g) = Cu content (wt%)/63.55 × 1000.

MeOH conversion (C_MeOH_, %) = reacted methanol/introduced methanol × 100%.

DMC selectivity (S_DMC_, %) = 2 produced DMC/reacted methanol × 100%.

Turnover frequency = produced DMC/(the molar amount of copper × reaction time).

### Characterization

X-Ray diffraction (XRD) patterns were recorded on a Rigaku D-Max 2500 diffractometer, using Cu *K*α radiation (*λ* = 0.154 nm) at 40 kV and 100 mA, with a scanning rate of 4° min^−1^ at 2*θ* of 5°–85°. Transmission electron microscopy (TEM) analysis was carried out on a JEM 2100F field emission transmission electron microscope (JEOL, Tokyo, Japan) operating at 200 KeV. TEM samples were prepared by immersing C-coated Cu grids in ethanol solutions of samples and drying at room temperature. Thermogravimetric (TG) analysis was conducted on a thermogravimetric analyzer, STA 449 F3 Jupiter (NETZSCH), with a N_2_ or air flow rate of 50 mL/min. Surface areas and pore volumes were determined from nitrogen adsorption isotherms at 77 K using the 3H-2000PS2 (Beishide) surface area analyzer. The Brunauer-Emmett-Teller (BET) specific surface areas were calculated using adsorption data at relative pressure range of *P*/*P*_0_ = 0.04–0.3. Mesopore pore size distribution curves were calculated by the BJH (Barrett-Joyner-Halenda) method from the adsorption branch. The total pore volumes were estimated from the amount of nitrogen adsorbed at a relative pressure (*P*/*P*_0_) of 0.99. The copper content is determined by dissolving the catalyst in a strong acid mixture followed by analysis of atomic adsorption spectrometry (AAS) using SpectrAA-220 AAS equipment. The analysis of the reaction product was performed by gas chromatography (GC; Agilent 6890) using an FID detector.

## Results and Discussion

### Texture Parameters and Thermal Stability of As-prepared Support

**Fig. 1:**
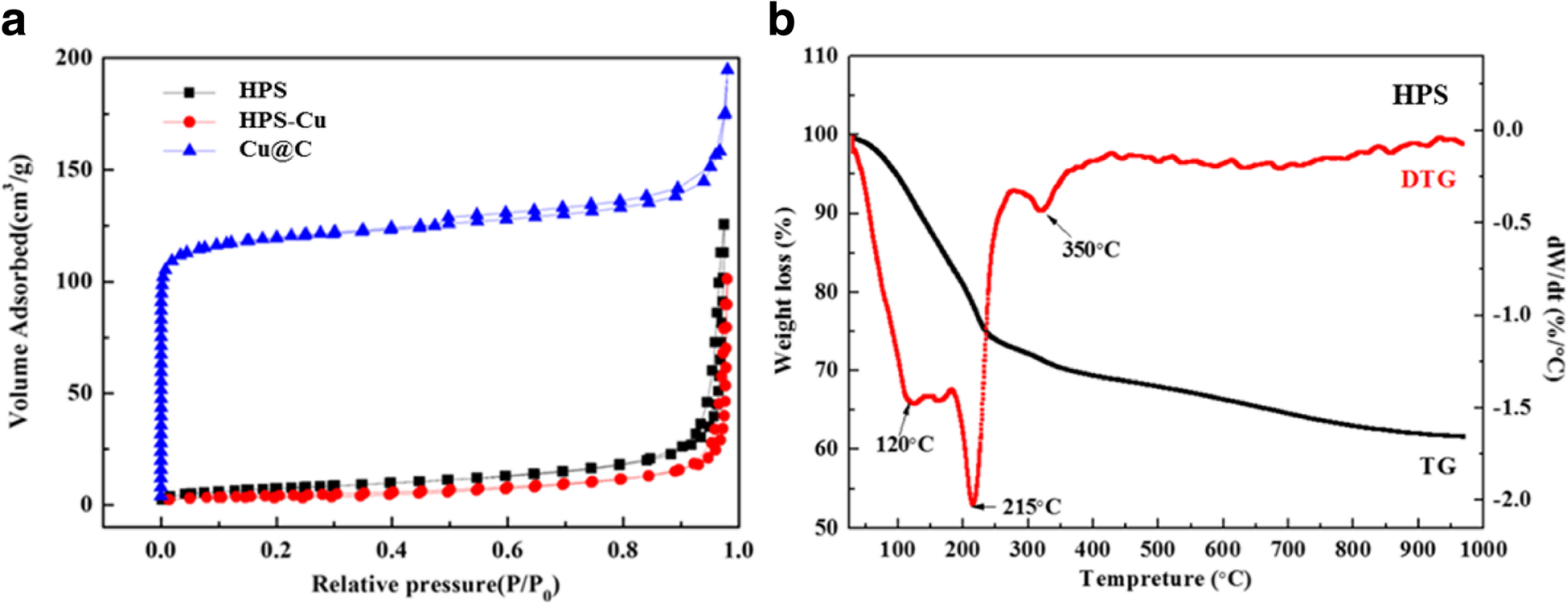
**a** N_2_ adsorption-desorption isotherm of the products obtained after each step: HPS, HPS-Cu^2+^, and Cu@C. **b** TG-DTG profiles of HPS

**Fig. 2:**
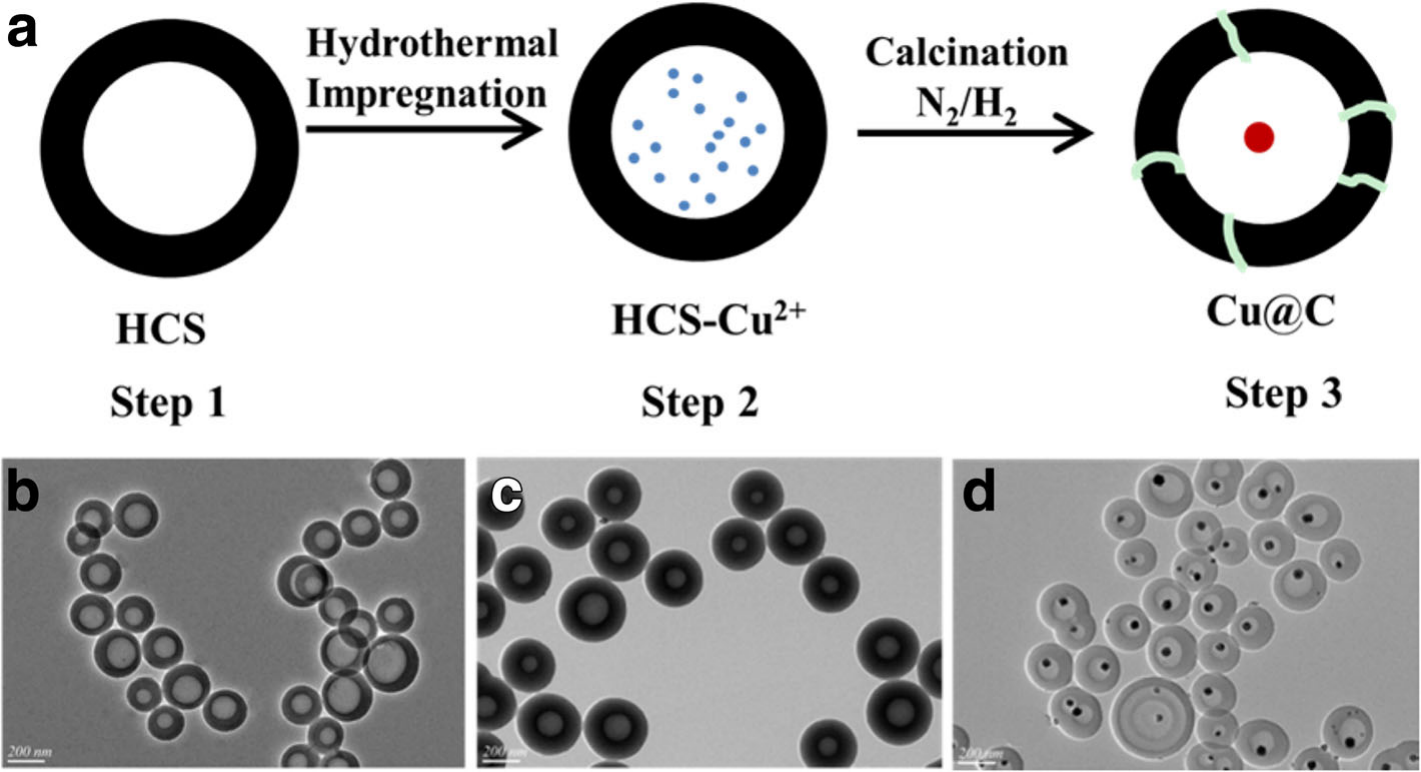
**a** Schematic illustration for the synthesis of Cu@C nanocomposites under hydrothermal impregnation condition. TEM images of the products obtained after each step: **b** HCS, **c** HCS-Cu^2+^, and **d** Cu@C

The BET surface area and pore volume of the samples involved in different preparation stages are summarized in Table 1. As seen in Fig. 1a and Table 1, the obtained HPS has low BET surface areas (~23 m^2^ g^−1^). So, it is hardly to pursue the introduction of the catalyst precursors through conventional impregnation method. Thus, we utilize hydrothermal impregnation process to enhance diffusion ability so that copper precursor can be successfully drawn into the cavity of HPS. The BET surface area of HPS and HPS-Cu changed from 23 to 15 m^2^ g^−1^ certified the statement. In addition, TEM images in Fig. 2 further confirm the Cu nanoparticles formed exclusively within the confines of the carbon shell.

**Table 1:** Textural parameters of the products obtained after each step: HPS, HPS-Cu, and Cu@C

Catalysts	S_BET_^a^ (m^2^ g^−1^)	V_T_^b^ (cm^3^ g^−1^)
HPS	23	0.19
HPS-Cu	15	0.16
Cu@C	370	0.30

^a^S_BET_ is calculated by the Brunauer-Emmet-Teller (BET) method

^b^V_T_ represents the total pore volume measured at P/P_0_ = 0.98

The carbonization process of the HPS is investigated by TG. Figure 1b shows the result of TG-DTG in N_2_. Throughout this entire interval, the major loss of HPS appears near 215 °C and is complete around 350 °C. This can be ascribed to the decomposition of oleic acid embedded inside HPS and carbonization of polymer framework [22]. Thus, compared with TG curves of Cu@C catalysts (see in Fig. 5b), to ensure the carbonation completely of HPS and prevent the Cu nanoparticles from aggregation, 400 °C was determined as the optimum preparation temperature.

### Structural Properties of Cu@C Nanocomposites

**Fig. 3:**
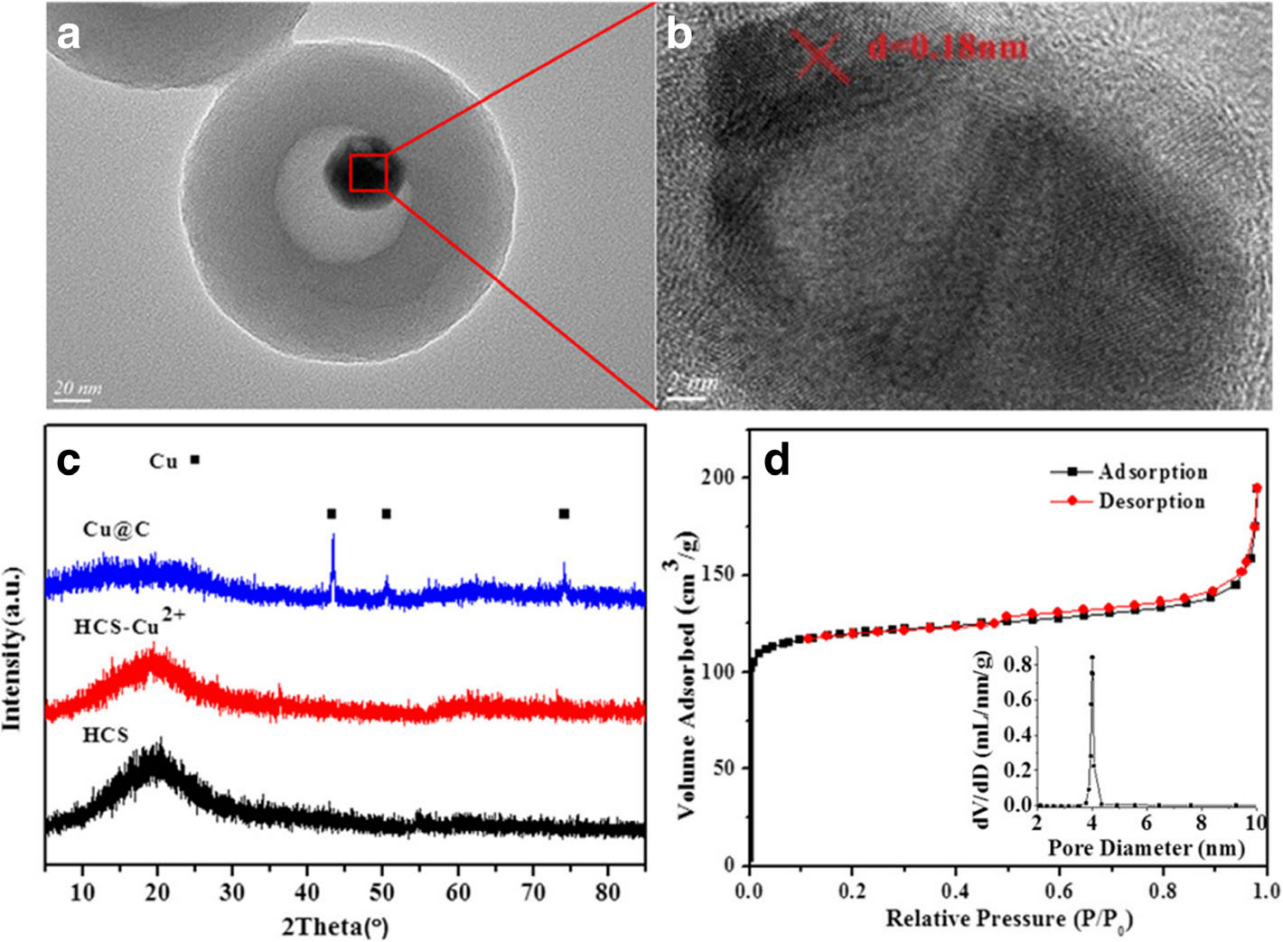
**a**, **b** TEM images of copper core in Cu@C-0.12. **c** XRD patterns of the products obtained after each step: HCS, HCS-Cu^2+^, and Cu@C-0.12. **d** N_2_ adsorption-desorption isotherm and distribution of pore size of Cu@C-0.12

Taken Cu@C-0.12 yolk-shell nanocomposite as example, the synthesis procedure for the preparation of yolk-shell structures with Cu NPs encapsulated by carbon shell, following a ship-in-a-bottle strategy, is illustrated in Fig. 2a. Figure 2b, c shows typical TEM images of the resulting product obtained in each step. As seen, HCSs with uniform size of about 210 nm have been successfully synthesized (Fig. 2b). During hydrothermal impregnation process, no obvious difference can be observed between HCSs and HCS-Cu^2+^ (Fig. 2c). However, after calcination, the hollow morphology is maintained, but Cu NPs can be observed due to the decomposition of copper salts. Finally, the yolk-shell-structured Cu@C (Fig. 2d) with a diameter of ~200 nm and cavity size of ~80 nm is achieved. High-resolution transmission electron microscopy (HRTEM) (Fig. 3b) shows that the core particles have a space of 0.18 nm indexed to the Cu (2 0 0) plane. This is consistent with the results of XRD (Fig. 3c), where diffraction peaks at 2*θ* = 43.3°, 50.4°, and 74.1° became observable because Cu^2+^ species on the precursor (HCS-Cu^2+^) are reduced to metal Cu under a reducing atmosphere, corresponding to the specific (1 1 1), (2 0 0), and (2 2 0) crystal planes of Cu, respectively, which is based on the JCPDS card 04-0836. The N_2_-adsorption-desorption isotherm of the resulting Cu@C-0.12 present a type I isotherm, demonstrating that there are abundant micropores on the carbon shells of Cu@C-0.12 (Fig. 3d). This sample has a BET surface area of 365 m^2^/g accompanied by a pore volume of 0.23 cm^3^/g. Low specific surface area together narrow microporosity are usually pointed out as main disadvantages, limiting their applications, which will be discussed below. Detailed texture parameters of samples are summarized in Table 2.

**Table 2:** Catalytic performance of Cu@C-X (X = 0.03, 0.06, 0.12, 0.24) catalysts with different Cu core sizes

Catalysts	W_catalyst_^a^ (g)	Cu particle size^b^ (nm)		S_DMC_ (%)	C_MeOH_ (%)	TOF (h^−1^)
		TEM	XRD			
Cu@C-0.03	1.6129	30.1	26.6	93.8	2.91	1.35
Cu@C-0.06	1.2712	40.2	35.9	94.1	3.55	1.65
Cu@C-0.12	1.1905	45.1	41.7	94.3	4.38	2.04
Cu@C-0.24	0.9615	55.1	52.2	92.5	3.13	1.43

Reaction Conditions: C_Cu_ = 150 mmol/L, *T* = 120 °C, initial pressure = 3.0 MPa (P_CO_:P_O2_ = 2:1), *t* = 120 min, stirring speed = 750 rpm, V_MeOH_ = 10 mL

^a^The weight of catalyst

^b^The size of Cu cores

The mechanistic pathway for the formation of a single Cu NPs within the carbon shell can be explained by the confined nucleation-and-growth process. In the pyrolysis progress, many initial tiny CuO nuclei formed and distributed completely within the hollow cavity due to the decomposition of the incorporated Cu(NO_3_)_2_ molecules. When the reducing agent H_2_ diffuses into the cavity, the formed CuO nuclei are further reduced to metallic Cu nuclei, which tend to migrate and aggregate to form bigger particles. Once the larger ones form, the remaining Cu nuclei within the cavity will be successively absorbed onto the surface of the preformed particles, which results in the growth of Cu nanocrystal. Similar mechanism also has been proposed elsewhere [23]. Based on the nucleation-and-growth process, it could be inferred that the size of the resulting Cu core can be controlled by adjusting the amount of copper salt precursor accommodated in the confined cavity.

### Size Control of Cu Core

**Fig. 4:**
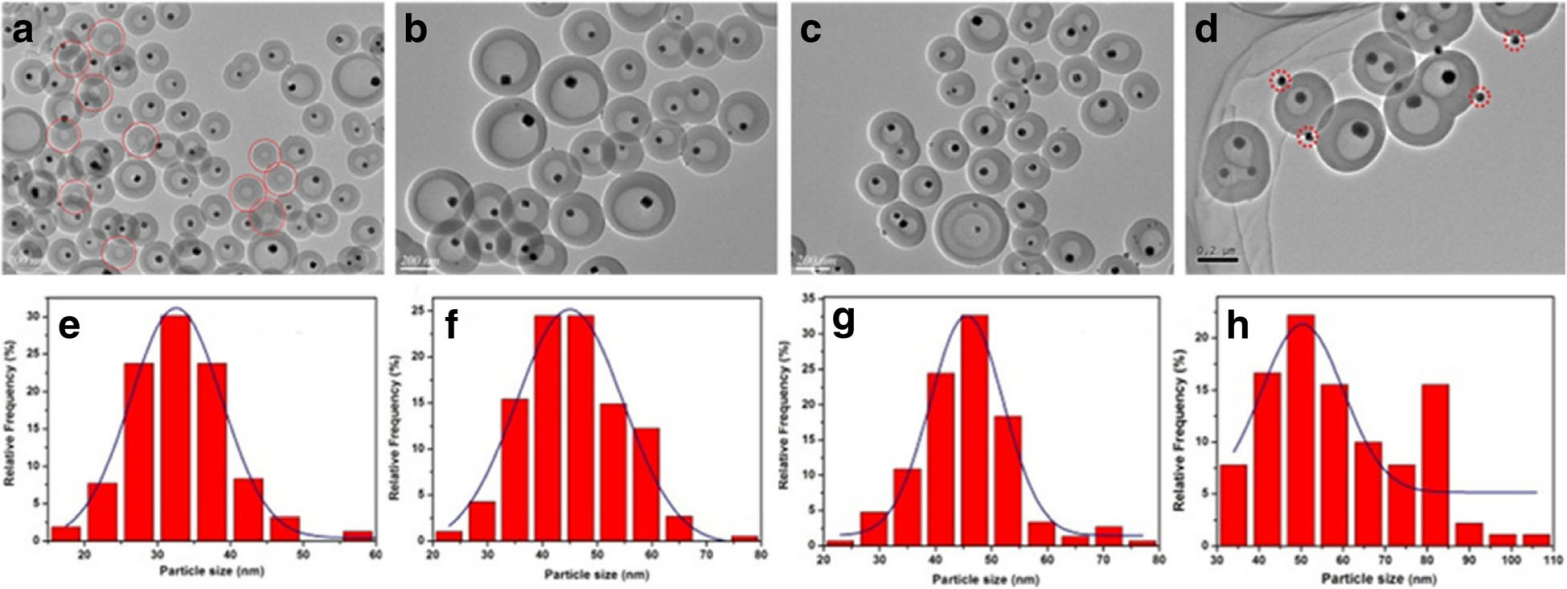
TEM images and corresponding size distribution histograms of Cu NPs in samples: **a**, **e** Cu@C-0.03, **b**, **f** Cu@C-0.06, **c**, **g** Cu@C-0.12, and **d**, **h** Cu@C-0.24

By varying Cu(NO_3_)_2_ concentrations from 0.03 to 0.24 M, a series of yolk-shell nanocomposites, denoted as Cu@C-X (X = 0.03, 0.06, 0.12, 0.24), were obtained. The morphology and size of the products were examined by TEM. As seen in Fig. 4a–d, almost all of the hollow nanospheres consist of a single particle inside. However, the Cu core size of the resultant nanospheres increases from 30 ± 1.3 to 55 ± 2.5 nm (Fig. 4e–h) with an increasing Cu(NO_3_)_2_ concentrations, as determined from TEM images by taking at least 150 particles into account. Notably, a fraction of hollow carbon spheres (HCSs) are coexistent with YSNs for the low Cu(NO_3_)_2_ concentration (Fig. 4a). Besides, a few small Cu NPs decorated on the outer surface of the carbon shell (Fig. 4d), which could be caused by the decomposition and aggregation of the residual Cu(NO_3_)_2_ outside the shell.

**Fig. 5:**
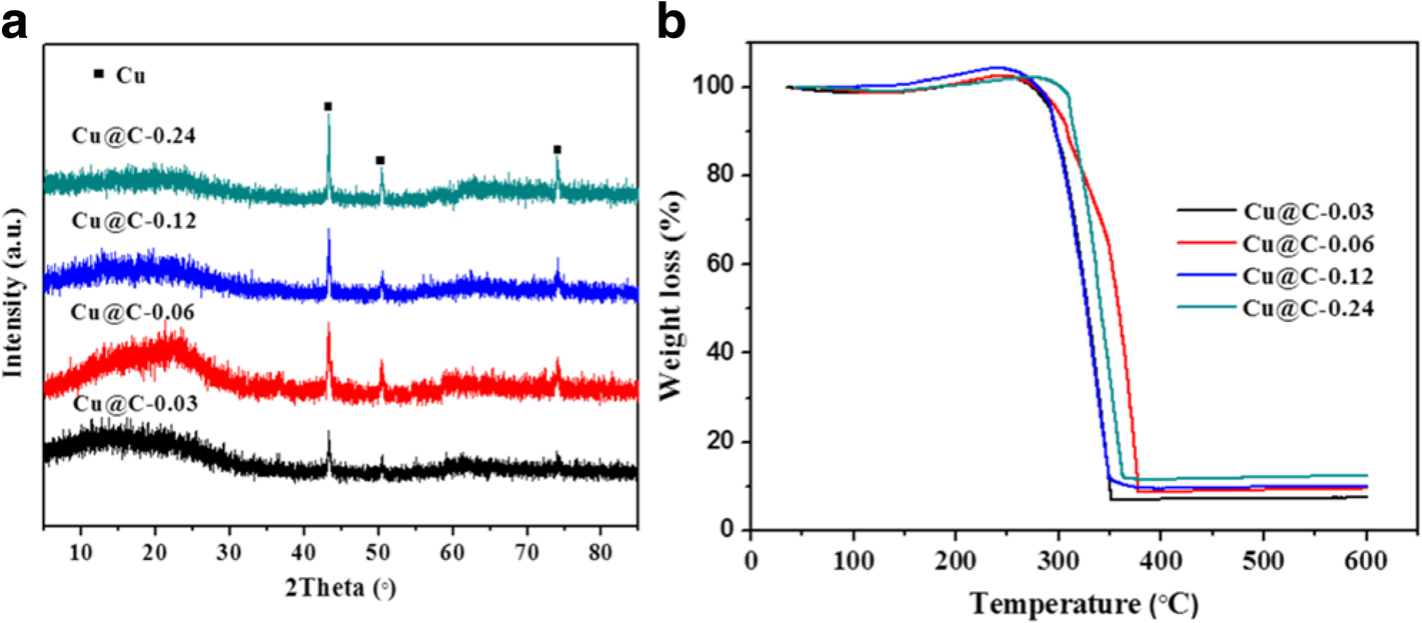
**a** XRD patterns and **b** TG curves of samples: Cu@C-0.24, Cu@C-0.12, Cu@C-0.06, Cu@C-0.03

Figure 5a shows the XRD patterns of as-prepared Cu@C-X (X = 0.03, 0.06, 0.12, 0.24). All the samples present three typical reflection peaks indexed to Cu crystals (JCPDS Card No. 04-0836). As the copper salt concentration increases, the diffraction peaks are much stronger and sharper, while the Cu NP size increases from 26.6 to 52.2 nm by using the Scherrer equation based on the strongest peak of the patterns, which is in well agreement with the TEM results. Furthermore, TG analysis was conducted to determine the Cu content of the nanospheres in Fig. 5b. Assuming that the residues consist of completely CuO, the Cu loading amounts of Cu@C-X (X = 0.03, 0.06, 0.12, 0.24) are calculated to be approximately 5.9, 7.5, 8.0, and 9.9 wt%, respectively, which is identical to the values determined from the AAS analysis.

### Catalytic Performance of Cu@C-X (X = 0.03, 0.06, 0.12, 0.24)

The as-prepared Cu@C catalyst was tested for the liquid-phase oxidative carbonylation of methanol to DMC (Table 2). Unexpectedly, although being better than others, the Cu@C-0.12 catalyst only gave an extremely inefficient methanol conversion of 0.82%. The low catalytic activity could be associated with the lack of sufficient porosity and large pore volume in the shell. To the best of our knowledge, pores located on the shell acts as channels connecting the void of the spheres with the external environment [24]. Although the shell thickness of Cu@C-0.12 is ~15 nm, the lack of sufficient porosity (the structural pore volume is 0.23 cm^3^/g with a low specific surface area of 365 m^2^/g) constrains the amount of reactant molecules to diffuse into the cavities and further to contact the buried active component of Cu cores. Thus, it is critical to create more porosity in the shells to facilitate the mass transport. As known, KOH activation is a well-established method in adjusting the porosity of carbon materials [25–27]. With this method, micropores and mesopores can be introduced into carbon, along with a significant increase in specific surface area and pore volume [28]. During the activation procedure, KOH amount is generally considered as a critical factor to influence the porous structure; thus, different mass ratios of KOH/HCS have been made to optimize the activated Cu@C-0.12.

### Physicochemical Properties of Cu@A-HCS

**Fig. 6:**
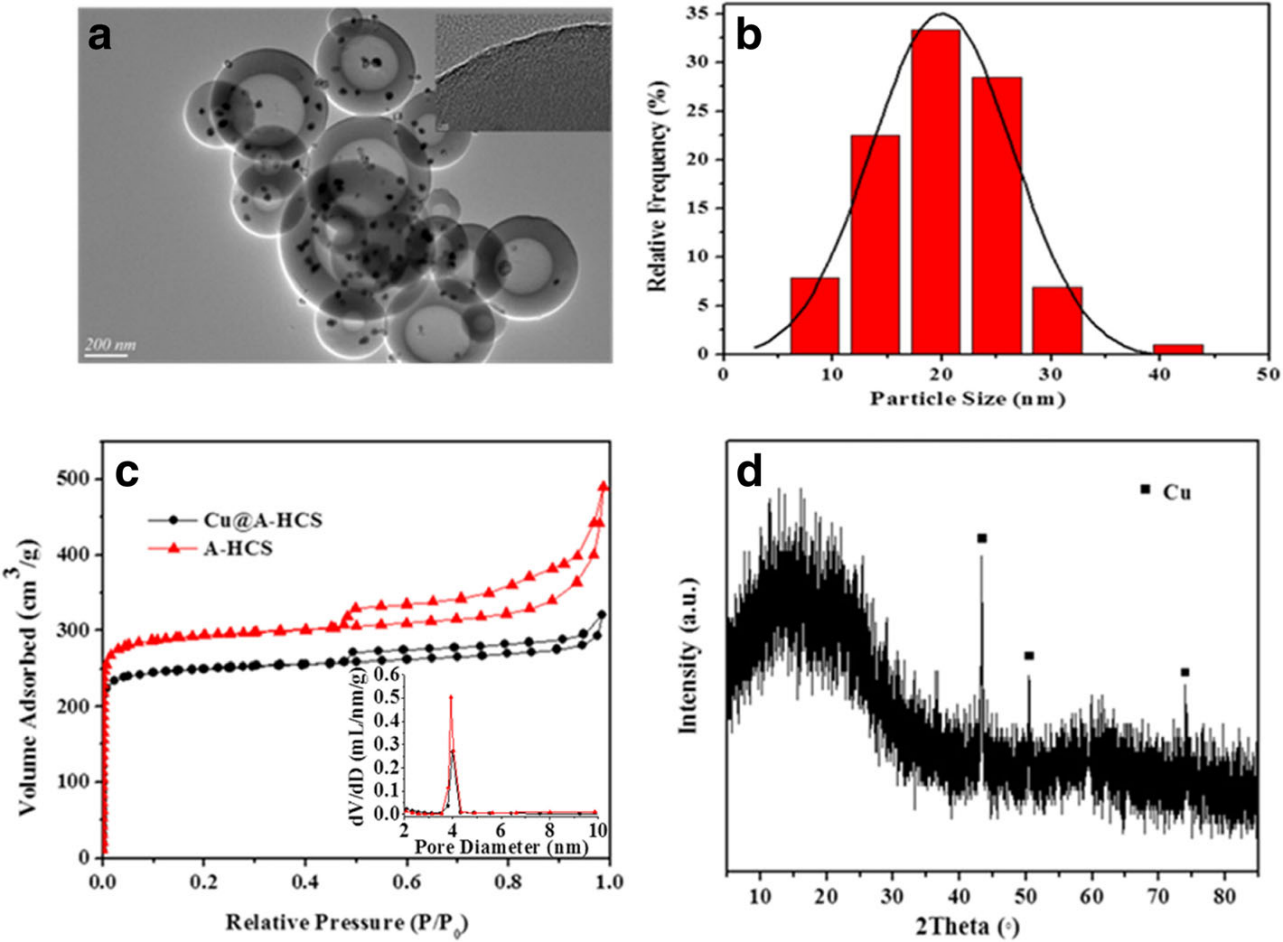
**a** TEM image of Cu@A-HCS and **b** its corresponding Cu particle size. **c** N_2_ adsorption-desorption isotherms and distributions of pore size of A-HCS and Cu@A-HCS. **d** XRD patterns of Cu@A-HCS catalyst

TEM image (Fig. 6a) shows that the activated Cu@A-HCS sample maintains the spherical morphology at the lower mass ratio of KOH/HCS (1/4), but partly or severely etched with KOH/HCS mass ratio higher than 1/2 (See the Supporting Information Fig. 2a, b). This result is in good agreement with previous reports that excessive amount of KOH will lead to more carbon burn-off and destroy the morphology [29]. Interestingly for Cu@A-HCS nanocomposites, after activation, highly dispersed copper particles are predominantly embedded in the shell of the hollow spheres, which coexist with several ones encapsulated in the cavities. When compared to Cu@C-0.12, the Cu NPs insert the shell display the relatively smaller particle size centered at 18 ± 2 nm (Fig. 6b) because the shell matrix stops the small Cu clusters from growing larger. The existence of white dots in the shell suggests the existence of disordered microspores. Figure 6c shows the N_2_-adsorption-desorption isotherms of the Cu@C-HCS, which exhibit representative type IV curves associated with mesopore feature, revealing that the activated samples possess hierarchically micro−/mesoporous structures. Also, it can be found that after KOH activation at 700 °C for 2 h, the surface area of A-HCS increased from 471 to 989 m^2^/g, even larger than activated carbon (812 m^2^/g), and the micropore volume (V_mic_), the mesopore volume (V_mes_), and the total volume (V_T_) also increased, but the ratio of V_mic_ to V_T_ tends to decrease. This result indicates that more mesopores are created after KOH activation, which is possibly related to the widening of micropores or the creation of mesopores by the presence of KOH [30]. The typical large surface area and developed porosity of Cu@A-HCS catalysts favor the dispersion of the active phase over the support, guarantee the fast transfer of matter between the confined catalyst and the external environment (reactants), and increase its resistance to sintering at high metal loading [31]. As confirmed by XRD pattern in Fig. 6d of Cu@A-HCS, all the peaks can be indexed undisputedly to cubic Cu (JCPDS 04-0836); meanwhile, the broadening of the characteristic peaks implies the formation of the Cu NPs with small size. Actually, the average size of the Cu NPs in Cu@A-HCS is estimated to be 15 nm according to the Scherrer equation, which is in agreement with the result obtained by TEM. As predict, the Cu@A-HCS catalysts with 11 wt% of Cu determined by AAS were obtained using the same method, higher than Cu@C-0.12. More importantly, during the activation process, oxygen-containing functional groups possibly originated from KOH activation are inevitably introduced in HCSs [27]. Overall, the generation of surface groups, the increase in surface area and pore volume, synergistically result in the highly dispersion of Cu NPs, which is beneficial to the promotion of the catalytic activity [32–34]. Detailed textural properties are summarized in Table 3.

**Table 3:** Textural parameters of HCS, Cu@C, and activated support A-HCS and catalyst Cu@A-HCS

Catalysts	S_BET_^a^ (m^2^/g)	D_BJH_^b^ (nm)	V_T_^c^ (cm^3/^g)	V_mic_^d^ (cm^3^/g)	V_Mic_/V_T_^d^ (%)
A-HCS	989	3.89	0.78	0.37	47
Cu@A-HCS	771	3.91	0.50	0.36	72
HCS	471	4.01	0.37	0.20	54
Cu@C-0.12	365	3.99	0.26	0.19	73

^a^The specific area (S_BET_) is calculated by the Brunauer-Emmet-Teller (BET) method

^b^D_BJH_ represents the pore size distribution calculated by the Barrett-Joyner-Halenda (BJH) method

^c^V_T_ represents the total pore volume measured at P/P_0_ = 0.98

^d^V_mic_ represents the volume of micropore calculated by t-plot method

### Catalytic Performance of Cu@A-HCS

The catalytic performances of A-HCS and Cu@A-HCS are summarized in Table 4. As shown in Table 4, it is clear that the support A-HCS exhibited no catalytic activity on synthesis of DMC. As expected, the catalytic properties of activated sample improved dramatically as compared to non-activated ones. It is noteworthy that compared to 2.04 h^−1^ and 4.38% for Cu@C-0.12, the incipient activity of Cu@A-HCS exhibits a near fourfold increase in the TOF of 8.6 h^−1^ accompanied by a correspondingly dramatically increase in C_MeOH_ of 17.1%, respectively under the same conditions. These remarkable results are reasonable in considering the surge in the surface area and the pore volumes of the carbon shells can positively adsorb more reactant molecules from the bulk solution, facilitate the diffusion rates through the channels significantly, and enrich them in the void space of the catalysts, resulting in a higher reactant concentration for accessible confined catalysis. A catalyst with reasonably long lifetime is critical to its application in industry. The catalyst chosen is activated Cu@A-HCS sample with a promising activity to test the durability in a batch system described above. Heterogeneous catalysts often suffer from a decrease of the activity as an extensive leaching of active metal species during reactions [35]. And equally important is the stability against coalescence for nanocrystal-based catalyst [36]. In our case, as summarized in Table 3, the recovered Cu@A-HCS catalyst (separated by filtration) maintains much higher catalytic activity than CuCl even after seven runs (entries 2-8), and the average leaching of Cu, which is the active constituent of the catalyst, is around 0.004%, remaining nearly the same to the fresh one. Meanwhile, the crystal structure and morphology of the catalysts did scarcely not change after successive cycles (Additional file 1: Figure S2). Apparently, the presence of the porous carbon shell is sufficient for stabilizing the active metal species by preventing their aggregation and leaching; at the same time, the shells are permeable enough so that catalytic surfaces remain accessible and advantageous to the reactants and products [12]. Hence, the YSN catalysts are effective and noncorrosive catalytic systems, where the Cu NPs as core materials encapsulated in the cavity of HCSs afford reactive centers, and the porous carbon shell prevents the core from aggregation and leaching under reaction conditions.

**Table 4:** Results for recycling of activated Cu@A-HCS catalysts activated with KOH

No.	Catalysts	W_catalyst_^a^ (g)	C_Cu_^b^ (mmol/L)	S_DMC_ (%)	C_MeOH_ (%)	TOF (h^−1^)
1	A-HCS	0.8700	–	–	–	–
2	Cu@A-HCS	0.8672	150	91.2	17.1	8.6
3	Removed from no.2	0.8551	148	91.3	17.3	8.8
4	Removed from no.3	0.8436	146	90.8	17.2	8.8
5	Removed from no.4	0.8263	143	91.4	17.1	9.0
6	Removed from no.5	0.8147	141	91.3	17.0	9.1
7	Removed from no.6	0.7974	138	91.1	17.0	9.2
8	Removed from no.7	0.7827	136	91.5	16.9	9.4

Reaction conditions: *T* = 120 °C, initial pressure = 3.0 MPa (P_CO_:P_O2_ = 2:1), *t* = 120 min, stirring speed = 750 rpm, V_MeOH_ = 10 mL

^a^The weight of catalyst

^b^The concentration of copper in the slurry system

## Conclusions

In summary, we have presented a facile ship-in-a-bottle strategy for the fabrication of yolk-carbon shell nanostructures composed of Cu NPs with tailored size in narrow distributions by adjusting the concentration of copper salts. As demonstrated, the catalytic properties of this rattle-type system in oxidative carbonylation of methanol to DMC are highly porosity dependent. Activated sample with extremely high surface area enables the creation of highly efficient confined nanoreactors for catalytic reactions with considerable higher conversion (17.1%) and TOF (8.6 h^−1^), long lifetime, and negligible leaching in each cycle, which unquestionably satisfy the clean production of green chemical DMC. Moreover, the synthesis route described in this paper may open up new opportunities for preparing yolk-shell nanostructures with various compositions confined within the carbon shell.

## Additional File

Additional file 1: Figure S1. TEM images of samples with different mass ratios of KOH/HCS: (a) 1:1, (b) 1:2. Figure S2. (a) TEM image and (b) XRD pattern of Cu@A-HCS catalyst after seven runs. (DOCX 469 kb)
