# Supplementary material for: Fabrication of Yolk-Shell Cu@C Nanocomposites as High-Performance Catalysts in Oxidative Carbonylation of Methanol to Dimethyl Carbonate
Source: Nanoscale Res Lett. 2017 Aug 8;12:481. doi: 10.1186/s11671-017-2258-7 (PMC5548704; doi:10.1186/s11671-017-2258-7)
Supplement: Additional file 1: Figure S1. — TEM images of samples with different mass ratios of KOH/HCS: (a) 1:1, (b) 1:2. Figure S2. (a) TEM image and (b) XRD pattern of Cu@A-HCS catalyst after seven runs. (DOCX 469 kb) [file 11671_2017_2258_MOESM1_ESM.docx]

**Additional file 1**

**Fabrication of yolk-shell Cu@C nanocomposites as high-performance catalysts in oxidative carbonylation of methanol to dimethyl carbonate**

Juan Wang, Panpan Hao, Leilei Yang, Ruina Shi, Shusen Liu, Jun Ren*, Zhong Li

Key Laboratory of Coal Science and Technology (Taiyuan University of Technology),

Ministry of Education and Shanxi Province, Taiyuan 030024, China

*Corresponding author. Mailing address for correspondence: No. 79 Yingze West Street, Taiyuan 030024, China. Tel/Fax: +86 351 6018598.

E-mail address: renjun@tyut.edu.cn (J. Ren).


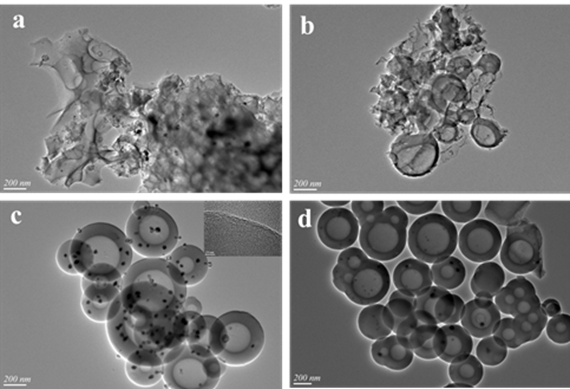


Fig. S1. TEM images of samples with different mass ratios of KOH/HCS: (a) 1:1, (b) 1:2


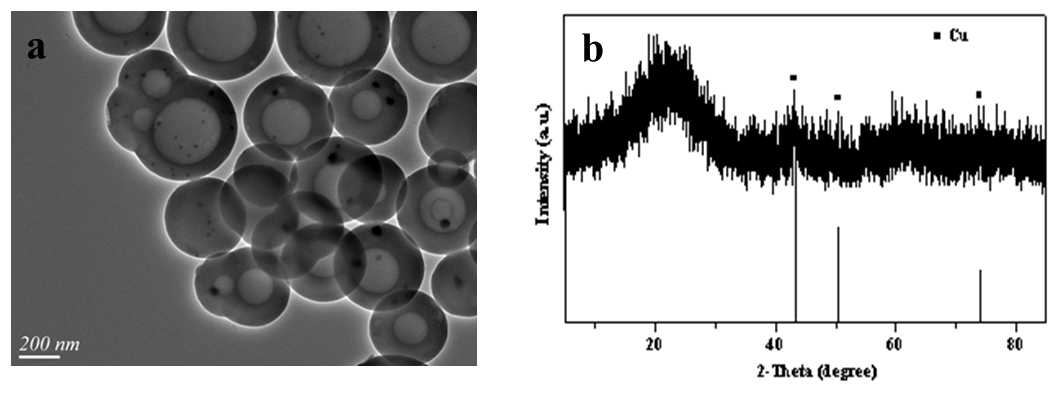


Fig. S2. (a) TEM image and (b) XRD pattern of Cu@A-HCS catalyst after seven runs.
